# London Surgeons and Hospitals: Operations for Dyspareunia and Pruritis—Hepatic Cysts—Hunter’s Museum—Obstetrical Society: Discussion of Priestley’s Paper

**Published:** 1885-11

**Authors:** C. C. F. Gay

**Affiliations:** London, Eng.


### Foreign Correspondence.

LONDON SURGEONS AND HOSPITALS: FACILITIES FOR STUDENTS—OPERATIONS FOR DYSPAREUNIA AND PRURITIS—HEPATIC CYSTS—HUNTER'S MUSEUM—OBSTETRICAL SOCIETY: DISCUSSION OF PRIESTLEY'S PAPER.

LONDON, Eng., September, 1885.

*Messrs. Editors :*

I have seen much surgery and witnessed many operations of several surgeons during my brief sojourn in London. I find naught to criticise, but much to commend; yet I must say that, so far as my observation extends, the surgeons here are in nowise superior to those of my own country. The advantage to be derived by students and practitioners in coming to London, consists in the abundance of clinical material furnished by so many hospitals.

One may keep constantly employed every day of the week, provided he is supplied with the means of *entrée* to the hospitals in witnessing operations. There are, however, operation days—usually Wednesdays and Saturdays. On other days, one may be employed in walking through the wards with the visiting surgeon, and thus be able to see a great variety of most interesting cases, and find out, if he did not know before, how puzzling is the study of diagnosis in some of the patients, even to the most experienced and skillful surgeon.

As you are aware, I did not come abroad for the purpose of study, but for rest, recreation and in pursuit of health, which had become impaired by gratuitous service rendered at one of our public institutions. In some way to me unknown, but suspected only, poison had entered my system, and, for a time, threatened to undermine a hitherto strong and vigorous constitution. The finger had received a scratch by spicula of dead bone in an ex-section, and afterwards thrust into pus depots. I

had exposed myself to the toxic influence of malignant diphtheria while operating for tracheotomy; and last, but not least, and probably the most efficient cause, was the inhalations, for half an hour, of carbolic acid spray, so strongly prepared that breathing and speaking became difficult. One of these causes, singly or all combined, occurring very near together, and which were followed by chills, placed me *hors de combat.*

Feeling so much better after a sea voyage, I found myself able to perform much labor without fatigue; therefore, in a little time following, my taste and inclination was drawn into the congenial occupation of visiting hospitals and seeing everything within reach connected with my profession, and then, the next thing in order was the disposition to give your readers some account of what I saw.

At the Women's Hospital, I saw an operation for dyspareunia. The patient had been married three years; coitus was so painful that it could not be accomplished. On examination, the os was found quite occluded. The first step of the operation was to dilate the cervical canal with metallic sounds, until a number 12 was passed; next, strips of mucous membrane at the vulva and around the vaginal orifice, and extending toward the os, were removed by the knife, and this was followed by light touches of Pasqualle's porte caustique, and the vaginal canal packed by lint.

Another patient was a case of pruritus, so severe that the woman was quite willing to subject herself to an operation. The operation was, in some respects, similar to the preceding. The folds of mucous tissue were cut away and the denuded parts cauterized, and the cervical canal dilated with large gutta percha sounds. I am sorry not to be able to give the result of these operations.

It is a long "bus" ride out to the London Hospital, and when one reaches it, he will be reminded of an alms-house; the atmosphere does not seem the most wholesome. There are between seven and eight hundred beds; provision is made to receive all who may knock at its doors ; none are turned away. It is a *free* hospital, and, of course, furnishes much clinical material for study.

Fenwick, whom I was under engagement to meet at a certain hour, was kind enough to show me many cases, some of which were of great interest. There were two convalescent patients of hepatic cyst. Diagnosis had been made certain and the cysts tapped and evacuated, with the result just above recorded. Operations were witnessed having no special importance, if I except two cases, an account of which I may be pleased to give at another time.

Located near King's College Hospital, is the Museum of the College of Surgeons. A visit here is of more interest to the surgeon than a visit to the British Museum. One may spend a full day here, and at its close will wonder how he came to forget his lunch. This museum contains Hunter's anatomical specimens, and the collection is very large.

The visitor will be first attracted by a fine bust of Hunter, which is conspicuously placed. The specimens of the museum are catalogued and numbered, so that scarcely any time is lost in looking for specimens. I wished to examine specimens of fracture of the acetabulum—a subject which had been quite recently made a study by your correspondent. Taking the catalogue in hand, I immediately found the case in which the bones were arranged in order, and was permitted to make critical inspection of them.

An entire letter might easily be made up of what I saw at this museum, and which I can but think would be of interest to your readers ; but I must not weary them. There are two skeletons standing, or hanging, side by side, worthy of mention on account of their height. One is said to represent a young man of 28 years, who died from intemperance. The skeleton measures seven and a half feet ; by the side of this one is another, comparatively short, only measuring six feet and seven inches.

The curator of the entomological department is anxious to procure a Colorado bug for his museum. I am under promise to ask the custodian of the Society of Natural Sciences to send him a bug.

Most of the societies of this city had adjourned for the summer before my arrival. I attended a session of the Obstetrical Society, at which there were present West, Playfair, Duncan and Lister; the latter attended, I suppose, by invitation, as the subject of the paper of the evening pertained to Listerism, read by Priestley, on the sanitary condition of the hospitals of Copenhagen and hospitals of Russia, which had been recently visited by the author of the paper. A spirited and very able discussion followed the reading of the paper, participated in by Duncan and others.

Duncan declared that the author and promoter of antisepsis should be crowned with greater honor than the discoverer of vaccination, since a larger amount of disease was prevented, and more lives saved by the former preventive method than by the latter. Variola appeared from time to time as an epidemic, while puerperal fever was constantly occurring everywhere in the world, and heretofore more lives were lost by this malady than were lost by both small-pox and cholera. He asserted that the mortality from puerperal fever was less in the hospitals where the antiseptic method was practiced, than in private practice where the method was not employed.

One gentleman who is connected with a lying-in hospital affirmed that in that institution but one case of puerperal fever had occurred in the last eleven hundred cases, and passed a high eulogium upon Lister and Listerism.

But I am reminded to close, lest my letter become wearisome to those who may chance to read it across the sea.

Yours truly,

C. C. F. G.

